# Oxytocin Receptor Polymorphism Is Associated With Sleep Apnea Symptoms

**DOI:** 10.1210/jendso/bvae198

**Published:** 2024-11-26

**Authors:** Hisanori Goto, Yasuhiko Yamamoto, Hiromasa Tsujiguchi, Takehiro Sato, Reina Yamamoto, Yumie Takeshita, Yujiro Nakano, Takayuki Kannon, Kazuyoshi Hosomichi, Keita Suzuki, Masaharu Nakamura, Yasuhiro Kambayashi, Jiaye Zhao, Atsushi Asai, Koji Katano, Aya Ogawa, Shinobu Fukushima, Aki Shibata, Fumihiko Suzuki, Hirohito Tsuboi, Akinori Hara, Mitsuhiro Kometani, Shigehiro Karashima, Takashi Yoneda, Atsushi Tajima, Hiroyuki Nakamura, Toshinari Takamura

**Affiliations:** Department of Endocrinology and Metabolism, Kanazawa University Graduate School of Medical Sciences, Kanazawa, Ishikawa 920-8640, Japan; Department of Biochemistry and Molecular Vascular Biology, Kanazawa University Graduate School of Medical Sciences, Kanazawa, Ishikawa 920-8640, Japan; Department of Biochemistry and Molecular Vascular Biology, Kanazawa University Graduate School of Medical Sciences, Kanazawa, Ishikawa 920-8640, Japan; Department of Hygiene and Public Health, Faculty of Medicine, Institute of Medical, Pharmaceutical and Health Sciences, Kanazawa University, Kanazawa City 920-8640, Japan; Advanced Preventive Medical Sciences Research Center, Kanazawa University, Kanazawa, Ishikawa 920-8640, Japan; Department of Human Biology and Anatomy, Graduate School of Medicine, University of the Ryukyus, Nishihara, Okinawa 903-0215, Japan; Department of Endocrinology and Metabolism, Kanazawa University Graduate School of Medical Sciences, Kanazawa, Ishikawa 920-8640, Japan; Department of Endocrinology and Metabolism, Kanazawa University Graduate School of Medical Sciences, Kanazawa, Ishikawa 920-8640, Japan; Department of Endocrinology and Metabolism, Kanazawa University Graduate School of Medical Sciences, Kanazawa, Ishikawa 920-8640, Japan; Department of Biomedical Data Science, School of Medicine, Fujita Health University, Toyoake, Aichi 470-1192, Japan; Laboratory of Computational Genomics, School of Life Science, Tokyo University of Pharmacy and Life Sciences, Hachioji, Tokyo 192-0392, Japan; Advanced Preventive Medical Sciences Research Center, Kanazawa University, Kanazawa, Ishikawa 920-8640, Japan; Department of Hygiene and Public Health, Faculty of Medicine, Institute of Medical, Pharmaceutical and Health Sciences, Kanazawa University, Kanazawa City 920-8640, Japan; Department of Hygiene and Public Health, Faculty of Medicine, Institute of Medical, Pharmaceutical and Health Sciences, Kanazawa University, Kanazawa City 920-8640, Japan; Department of Public Health, Faculty of Veterinary Medicine, Okayama University of Science, Imabari, Ehime 794-8555, Japan; Department of Hygiene and Public Health, Faculty of Medicine, Institute of Medical, Pharmaceutical and Health Sciences, Kanazawa University, Kanazawa City 920-8640, Japan; Advanced Preventive Medical Sciences Research Center, Kanazawa University, Kanazawa, Ishikawa 920-8640, Japan; Advanced Preventive Medical Sciences Research Center, Kanazawa University, Kanazawa, Ishikawa 920-8640, Japan; Department of Hygiene and Public Health, Faculty of Medicine, Institute of Medical, Pharmaceutical and Health Sciences, Kanazawa University, Kanazawa City 920-8640, Japan; Department of Hygiene and Public Health, Faculty of Medicine, Institute of Medical, Pharmaceutical and Health Sciences, Kanazawa University, Kanazawa City 920-8640, Japan; Department of Hygiene and Public Health, Faculty of Medicine, Institute of Medical, Pharmaceutical and Health Sciences, Kanazawa University, Kanazawa City 920-8640, Japan; Department of Hygiene and Public Health, Faculty of Medicine, Institute of Medical, Pharmaceutical and Health Sciences, Kanazawa University, Kanazawa City 920-8640, Japan; Department of Geriatric Dentistry, Ohu University School of Dentistry, Koriyama, Fukushima 963-8611, Japan; Department of Hygiene and Public Health, Faculty of Medicine, Institute of Medical, Pharmaceutical and Health Sciences, Kanazawa University, Kanazawa City 920-8640, Japan; Graduate School of Human Sciences, The University of Shiga Prefecture, Hikone, Shiga 522-8533, Japan; Department of Hygiene and Public Health, Faculty of Medicine, Institute of Medical, Pharmaceutical and Health Sciences, Kanazawa University, Kanazawa City 920-8640, Japan; Advanced Preventive Medical Sciences Research Center, Kanazawa University, Kanazawa, Ishikawa 920-8640, Japan; Department of Health Promotion and Medicine of the Future, Kanazawa University Graduate School of Medical Sciences, Kanazawa 920-8640, Japan; Institute of Liberal Arts and Science, Kanazawa University, Kanazawa 920-1192, Japan; Department of Health Promotion and Medicine of the Future, Kanazawa University Graduate School of Medical Sciences, Kanazawa 920-8640, Japan; Department of Bioinformatics and Genomics, Graduate School of Advanced Preventive Medical Sciences, Kanazawa University, Kanazawa, Ishikawa 920-8640, Japan; Department of Hygiene and Public Health, Faculty of Medicine, Institute of Medical, Pharmaceutical and Health Sciences, Kanazawa University, Kanazawa City 920-8640, Japan; Advanced Preventive Medical Sciences Research Center, Kanazawa University, Kanazawa, Ishikawa 920-8640, Japan; Department of Endocrinology and Metabolism, Kanazawa University Graduate School of Medical Sciences, Kanazawa, Ishikawa 920-8640, Japan

**Keywords:** oxytocin, oxytocin receptor, obstructive sleep apnea, genetic association study

## Abstract

**Context:**

Oxytocin supplementation improves obstructive sleep apnea (OSA), and animal studies suggest involvement of oxytocin in respiratory control. However, the relationship between endogenous oxytocin signaling and human sleep status remains undetermined.

**Objective:**

In this study, we approached the contribution of the intrinsic oxytocin-oxytocin receptor (OXTR) system to OSA by genetic association analysis.

**Methods:**

We analyzed the relationship between *OXTR* gene polymorphisms and sleep parameters using questionnaire data and sleep measurements in 305 Japanese participants. OSA symptoms were assessed in 225 of these individuals.

**Results:**

The OXTR rs2254298 A allele was more frequent in those with OSA symptoms than in those without (*P* = .0087). Although total scores on the Pittsburgh Sleep Quality Index questionnaire did not differ between the genotypes, breathlessness and snoring symptoms associated with OSA were significantly more frequent in individuals with rs2254298 A genotype (*P* = .00045 and *P* = .0089 for recessive models, respectively) than the G genotype. A multivariable analysis confirmed these genotype-phenotype associations even after adjusting for age, sex, and body mass index in a sensitivity analysis. Furthermore, objective sleep efficiency measured by actigraph was not significantly different between genotypes; however, subjective sleep efficiency was significantly lower in the rs2254298 A genotype (*P* = .013) compared with the G genotype. The frequency of the A allele is higher in East Asians, which may contribute to their lean OSA phenotype.

**Conclusion:**

The OXTR gene may contribute to OSA symptoms via the respiratory control system, although it could be in linkage disequilibrium with a true causal gene.

Obstructive sleep apnea (OSA) is a highly prevalent breathing disorder characterized by episodes of breathing cessation during sleep, and its prevalence is expected to increase further with the global increase in obesity [1]. OSA is not only associated with poor quality of life, reduced work efficiency, and increased driving accidents [2, 3] but also with increased incidence of hypertension [4], type 2 diabetes [5], stroke [6], atrial fibrillation, heart failure, and coronary disease [7]. Despite this ever-increasing health burden caused by OSA, beneficial drug therapies against this disease are currently not yet available or approved.

The pathogenesis of OSA is formed by a complex combination of anatomical factors of maxillofacial morphology, obesity, and physiological characteristics of the upper airway collapse [8, 9]. In addition, respiratory regulatory systems are also involved in the pathogenesis of upper airway obstruction. OSA pathogenesis has been considered to be caused by the instability of the ventilatory drive from the medullary respiratory center and the loss of genioglossus muscle activity [10, 11].

Not only environmental factors but also genetic factors are involved in OSA pathogenesis [12], and genetic polymorphisms involved in the OSA development have been reported by genome-wide association study (GWAS) [13-17].

Oxytocin (OXT) is a nonapeptide produced by magnocellular neurons located in the paraventricular and supraoptic nuclei, secreted by the posterior pituitary gland, and acts as a hormone. It is also widely secreted into the brain through exocytosis from the cell bodies and dendrites of magnocellular neurons, where it plays an essential role in regulating social behaviors and cognitive processes [18, 19]. Furthermore, parvocellular OXTergic neurons in the paraventricular nuclei project to the medulla oblongata and spinal cord [20]. To date, OXT has been linked to several pathological conditions, and based on its usage in obstetrics, it is recognized for its high safety as a therapeutic agent [21]. Based on this background, its effectiveness as a treatment for various pathological conditions has been investigated. In clinical trials, the beneficial effects of nasal OXT administration on schizophrenia [22], pain [23], and acute alcohol withdrawal syndrome [24] have been reported. Of particular note is that intranasal administration of OXT was shown to have some efficacy in OSA [25, 26]. Drug targets in the pipeline with human genetic evidence of disease association are reported to be more than twice as likely to lead to an approved drug [27]. Therefore, this study aimed to clarify the association between the OXT-OXT receptor (OXTR) system and OSA from genetic association analysis, considering the therapeutic application of OXT.

The *OXTR* gene, which encodes the OXT receptor, a major player in OXT signaling, has been associated with various psychiatric disorders and social cognition through genetic association analysis [28]. In particular, the association between *OXTR* SNPs and diseases has been extensively studied, primarily in the field of autism. Among these, rs53576 and rs2254298 are the single nucleotide polymorphisms (SNPs) most frequently reported to be associated with autism and related phenotypes [29-35]. Although the neural circuits involved in OSA and psychiatric disorders, including autism, are different, rs53576 and rs2254298 have been consistently reported in large studies, suggesting their importance in influencing OXTR signaling. While most research on OXTR SNPs has focused on psychiatric disorders, these 2 SNPs have also been linked to a variety of conditions involving diverse organs and neural circuits, such as bowel disease [36], pain [37], alcohol abuse [38], and liver fibrosis [39]. These findings indicate that rs53576 and rs2254298 may have broader biological significance, potentially affecting biological systems including respiratory control.

To verify the involvement of endogenous OXT signals in sleep states, the relationship between genetic polymorphisms of *OXTR* and sleep-wake states measured by detailed sleep questionnaires, including OSA-related items and sleep measurements by actigraph, was investigated in a Japanese community population cohort.

## Methods

### Study Participants

**Figure 1.**
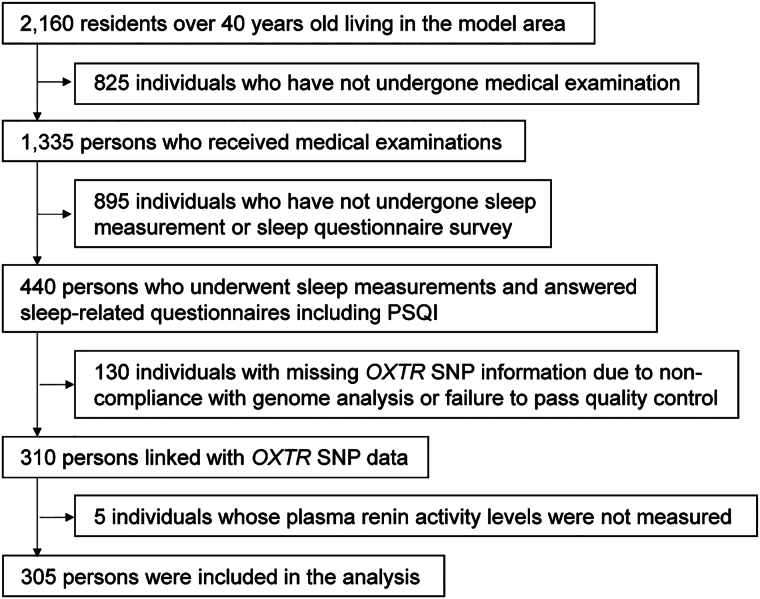
Flowchart of inclusion and exclusion criteria for the study population.

Data from a cross-sectional analysis known as the Shika study were obtained [40]. This project was conducted from 2013 to 2016 in the town of Shika, located in the rural sector of Ishikawa Prefecture, Japan. The primary objective was to monitor the health conditions of the local populace and explore preventive measures for lifestyle-related diseases. Cluster sampling, one of the random sampling methods, was employed as the sample extraction method. Specifically, this study was conducted on all middle-aged residents legally residing in 2 specific elementary school districts (Horimatsu Elementary School District and Higashi Masuho Elementary School District) within the town of Shiga, Ishikawa Prefecture. All 2160 residents were asked to complete a questionnaire and participate in a health check. All respondents who voluntarily agreed to participate in the health check were included in the study. From this group, 440 adults aged ≥ 40 years answered a comprehensive sleep questionnaire, including the Pittsburgh Sleep Quality Index (PSQI), and underwent sleep measurement using actigraphy, which determines sleep and wakefulness from time series data of measured physical activity. Of these, 130 individuals were excluded due to missing SNP information. Additionally, 4 individuals with missing plasma renin activity data were excluded, as plasma renin activity was used as an objective marker for the presence of OSA symptoms. Therefore, all analyses were conducted on the subset of participants with complete plasma renin activity data, to ensure consistency in the target population. Ultimately, the study analyzed 305 individuals. To enhance clarity, we have included a study flow diagram (Fig. 1) that clearly illustrates the participant selection process. OSA symptoms were defined based on observations of sleep apnea. Therefore, the association between OXTR SNPs and OSA symptoms was examined in the 225 subjects, excluding 80 subjects whose OSA status was unknown, likely due to the lack of third-party observation. To handle other missing data, pairwise deletion was utilized. Specifically, data were missing for 14 subjects for estimated glomerular filtration rate based on serum cystatin C (eGFRcys), 52 for nonspecific immunoglobulin E (IgE), 14 for N-terminal pro-brain-type natriuretic peptide (NT-proBNP), and 134 for alcohol intake as estimated by the brief-type self-administered diet history questionnaire (BDHQ).

### Data Collection

For each participant, data, including age, gender, and body metrics (height, weight), were obtained. The body mass index (BMI) was calculated by dividing an individual's weight in kilograms by the square of their height in meters. The alcohol consumption assessment leveraged the Japanese adaptation of the BDHQ, a validated instrument for determining the intake frequency of 58 prevalent Japanese food and beverage items in the last month, chosen for their representation in the National Health and Nutrition Survey of Japan. Designed for in-depth nutritional epidemiology studies in Japan, the BDHQ's reliability and repeatability have been verified in previous studies [41, 42]. Following overnight fasting, blood specimens were drawn from the forearm vein of the participants, typically between 08:00 and 12:00. These samples were thereafter forwarded to the SRL Kanazawa Laboratory and preserved at Kanazawa University at a temperature of −30 °C until the analysis.

To assess the presence of OSA symptoms, participants were asked in the questionnaire about “observed to stop breathing during sleep.” This question aligns with the “observed to stop breathing” item in the well-established STOP-Bang questionnaire, which is widely used for OSA screening [43]. The observation of cessation of breathing is one of the most effective items for OSA screening [44] and is a common component in many OSA screening questionnaires [45]. Participants who reported the “observed to stop breathing during sleep” were considered to have OSA symptoms. To assess sleep quality over the past month, a self-administered survey, the PSQI, consisting of 19 questions, was used [46]. These questions are designed to evaluate 7 distinct aspects of sleep: subjective sleep quality, sleep latency, sleep duration, subjective sleep efficiency, sleep disturbances, sleep medication use, and daytime activity dysfunction. The overall PSQI score is derived by summing the scores from these 7 areas, resulting in a total score ranging from 0 to 21. A lower score is indicative of better sleep quality. The PSQI is widely recognized as a reliable and valid tool for detecting sleep disorders and assessing sleep quality in various populations [47]. In addition, its usefulness as a screening tool for OSA has been reported [48]. The PSQI includes items related to snoring, which is also a component of the STOP-Bang score for OSA screening, as well as items related to breathlessness during sleep, which is associated with OSA. For sleep measurement, the ACOS MTN-220 actigraph was used to assess sleep/wake status. For data processing, we used an algorithm provided by the manufacturer. This algorithm determines sleep/wake states based on activity intensity data collected every 0.125 seconds and summarized over a 2-minute epoch. The algorithm employs a 5-variable linear model that includes activity intensity data for the current epoch, the 2 previous epochs, and the 2 subsequent epochs. The effectiveness of this algorithm has been demonstrated in previous studies, showing a high overall agreement with polysomnography [49].

### SNP Genotyping

The Qiagen QIAamp DNA Blood Maxi Kit was used to isolate the genomic DNA from the obtained blood samples. This process adhered to the manufacturer's guidelines or was alternatively executed by a specialized clinical lab, SRL Inc. The Japonica Array v2 produced by TOSHIBA Co., Ltd was used for genotyping of genome-wide SNPs. The SNP genotype data underwent rigorous quality control measures. Quality control procedures for both SNPs and participants involved several criteria: matching gender identity as indicated in karyotypic analysis with questionnaire results, call rate thresholds, evaluation through the Hardy–Weinberg equilibrium test, analysis of inbreeding coefficients, detection of cryptic familial relationships, and population structure assessment. This process and its protocols have been comprehensively described in previous publications [50]. The 1000Genomes_30x research data are the source of allele frequency data for the rs2254298 gene for each population [51].

### Statistical Analysis

All statistical analyses were performed using RStudio (version 2023.03.0 Build 386). The normality of continuous variables was assessed using both the Kolmogorov-Smirnov test and Q-Q plots. Results from the Q-Q plots were consistent with those from the Kolmogorov-Smirnov test. In our analysis of between-group differences, normally distributed continuous variables were evaluated using the 2-sample *t* test. The Mann-Whitney U test was employed for non-normally distributed variables. Categorical variables were evaluated using Pearson's chi-squared test. When 3 groups were compared, particularly under a codominant genetic model, the Kruskal-Wallis test was used for variables lacking normal distribution. Each SNP (rs2254298 and rs53576) was analyzed independently to assess their association with OSA symptoms. These 2 SNPs were selected based on their previous associations with psychiatric disorders and social behaviors, and importantly, they are not in significant linkage disequilibrium with each other, allowing for independent analyses. To account for multiple comparisons, a Bonferroni correction was applied based on the number of SNPs analyzed (2 SNPs: rs2254298 and rs53576) and the number of outcomes investigated after narrowing the analysis to rs2254298. For analyses involving the 2 SNPs, the corrected alpha level was set at 0.025 (0.05 ÷ 2). After focusing on rs2254298, the correction was applied based on the 3 outcomes, and the corrected alpha level was set at 0.0167 (0.05 ÷ 3). This ensured appropriate control of the type I error rate across all comparisons. Our approach to identifying the most appropriate genetic model followed the methodology outlined by Minelli et al [52] and modified by Rotman et al [53]. Univariate odds ratios (ORs) were calculated to contrast homozygous minor alleles with homozygous major alleles (represented as ORGG) and heterozygous with homozygous major alleles (represented as ORGg). The mode of inheritance was determined by the ratio of log ORGg to log ORGG, known as λ. Consistent with criteria from previous studies, λ values of < 0.25 were classified as recessive, between 0.25 and 0.75 as additive, and > 0.75 as dominant. In this study, after determining that the recessive model best fit the data, we focused solely on the recessive inheritance model for our analysis. For multivariable analysis, ordinal logistic regression and reported ORs were obtained along with their 95% CI.

### Ethics

Our study strictly complied with the ethical standards laid out in the Helsinki Declaration. The study protocol was approved by the Human Studies Ethics Committee of Kanazawa University Hospital, with approval numbers 1491, 2016-376. Furthermore, our study was officially registered in the University Hospital Medical Information Network (UMIN) Clinical Trials Registry, bearing the registration number UMIN 000024915. Before participation, written consent was obtained from all of the participants after being fully informed about the study.

## Results

First, to address and assess potential selection bias in the target population for which sleep and actigraph data were available, we compared sociodemographic variables and lifestyle factors between participants with and without sleep/actigraph data. Specifically, we assessed age, gender, BMI, smoking status (current, former, or nonsmoker), and alcohol consumption habits. The results of this comparison are shown in Supplementary Table S1 [54]. A statistically significant difference between the 2 groups was found in smoking status. Fewer participants with sleep data were current smokers, suggesting that sleep data might be collected from a more health-oriented group.

Table 1 details the background of patients analyzed in this study. The presence of OSA symptoms, frequency of sleeping medication use divided into 4 levels, subjective sleep quality assessed by the PSQI, and primary sleep parameters measured by actigraph were assessed. Since alcohol consumption significantly affect sleep quality [55], the amount of alcohol consumption assessed by the BDHQ was evaluated.

**Table 1.** Background, sleep parameters, and OXTR SNPs of the subjects

Parameter	n = 305
Age	64.00 [55.00, 70.00]
BMI	23.32 [21.31, 25.31]
Sex, n (%)	
Male	143 (46.9)
Female	162 (53.1)
OSA symptoms, n (%)	
No	189 (62.0)
Yes	36 (11.8)
Uncertain	80 (26.2)
Sleep medication, n (%)	
0	289 (94.8)
1	4 (1.3)
2	3 (1.0)
3	9 (3.0)
Alcohol intake (g/day)	2.39 [0.00, 17.85]
PSQIG	4.00 [3.00, 7.00]
Sleep parameters by actigraph	
Number of days measured by sleep monitor	7.00 [7.00, 7.00]
Total sleep time (min/day)	366.29 [322.67, 412.86]
Wake after sleep onset (min/day)	50.50 [26.44, 78.00]
Number of wakes after sleep onset (/day)	4.00 [2.17, 5.86]
Number of awakenings over 9 minutes (/day)	3.00 [0.14, 12.43]
Number of posture changes (/day)	16.38 [3.57, 55.14]
Sleep efficiency (%)	83.89 [76.07, 89.39]
Sleep latency (min)	14.57 [9.60, 22.50]
*OXTR* SNPs frequency	
rs53576 genotype (%)	
A/A	128 (42.0)
A/G	134 (43.9)
G/G	43 (14.1)
rs2254298 genotype (%)	
A/A	33 (10.8)
G/A	107 (35.1)
G/G	165 (54.1)

Categorical variables are displayed as n (%).

Continuous variables are displayed as mean (SD) or median [interquartile range].

Sleep medication is scored based on the frequency of sleep medication use in the last month as follows: 0, no use; 1, less than once per week; 2, 1-2 times per week; 3; more than 3 times per week.

Abbreviations: BMI, body mass index; OSA, obstructive sleep apnea; PSQIG, The Pittsburgh Sleep Quality Index Global score; SNP, single nucleotide polymorphism.

The number of SNPs that could be analyzed was first calculated due to the limited number of participants. Based on the allele frequency of rs2254298, a representative *OXTR* SNP, the proportion of patients with and without OSA symptoms, the number of alleles in the target population, and the Bonferroni-corrected α error, the number of SNPs available for analysis was calculated as 2. Two *OXTR* SNPs, rs2254298 and rs53576, were examined independently for their association with OSA symptoms because they have been most commonly reported to be associated with psychiatric disorders and social behaviors and are not in linkage disequilibrium with each other [29-35]. The A allele frequency of rs2254298 was significantly higher (*P* = .0087) in participants with OSA symptoms than in those without. With respect to rs53576, no significant difference was observed in allele frequency between patients with and without OSA symptoms (Table 2). Based on these results, subsequent analyses focused exclusively on rs2254298 to further explore its association with OSA-related symptoms.

**Table 2.** Association between respiratory arrest symptoms and rs2254298 allele frequency

SNP ID/Allele	Respiratory arrest symptoms		*P* value
rs2254298	No	Yes	
A	97 (25.7%)	30 (41.7%)	.0087*^a^*
G	281 (74.3%)	42 (58.3%)	.0087*^a^*

SNP ID/Allele	Respiratory arrest symptoms		*P* value
rs53576	No	Yes	
A	242 (64.0%)	52 (72.2%)	.23
G	136 (36.0%)	20 (27.8%)	

*P* values were from chi-square test.

^*a*^Significant difference (corrected α = 0.025, Bonferroni procedure).

Single nucleotide polymorphism (SNP) allele distribution significantly associates with sleep apnea severity, suggesting a genetic basis for its variability.

Factors associated with sleep quality and representative sleep indices were compared among genotypes of rs2254298 (Table 3). No significant differences were found between genotypes under recessive genetic model for items associated with sleep quality, such as age, sex, and frequency of sleep medication use. No significant differences were observed in the total PSQI scores or various sleep measurement indices assessed using actigraph, including objective sleep efficiency between genotypes (Table 3). Given that our study included 305 participants, it is possible that the lack of a significant association could be due to insufficient power.

**Table 3.** Association of rs2254298 genotype with factors affecting sleep quality and sleep metrics

Parameters		Genotypes of rs2254298			
		A/A	G/A	G/G	Recessive model
n		33	107	165	
Age		61.00 [49.00, 69.00]	64.00 [55.00, 70.00]	64.00 [56.00, 70.00]	0.24
BMI		22.51 [21.31, 24.61]	23.87 [21.55, 25.85]	23.12 [21.11, 24.86]	0.559
Sex (%)	Male	17 (51.5)	54 (50.5)	72 (43.6)	0.585
	Female	16 (48.5)	53 (49.5)	93 (56.4)	
	Frequency				
Sleep medication (%)	0	30 (90.9)	101 (94.4)	158 (95.8)	0.317
	1	2 (6.1)	1 (0.9)	1 (0.6)	
	2	0 (0.0)	1 (0.9)	2 (1.2)	
	3	1 (3.0)	4 (3.7)	4 (2.4)	
Alcohol intake (g/day)		4.18 [0.00, 24.10]	6.27 [0.00, 28.64]	0.23 [0.00, 12.81]	0.7
PSQIG		5.00 [3.00, 7.00]	5.00 [3.00, 7.00]	4.00 [3.00, 7.00]	0.23
Sleep parameters by actigraph					
Total sleep time (min/day)		344.00 [312.00, 388.00]	361.33 [310.41, 408.57]	376.57 [326.57, 420.00]	0.179
Wake after sleep onset (min/day)		49.43 [29.43, 78.57]	50.86 [28.57, 76.78]	50.50 [25.50, 78.29]	0.687
Number of wake after sleep onset (/day)		4.17 [2.43, 6.25]	4.00 [2.30, 5.67]	3.86 [2.00, 5.86]	0.621
Number of awakenings over 9 minutes (/day)		3.29 [1.86, 4.75]	2.86 [1.73, 4.43]	3.00 [1.67, 4.43]	0.665
Number of posture changes(/day)		16.71 [12.71, 20.50]	16.13 [11.29, 20.05]	16.71 [12.00, 22.71]	0.952
Sleep efficiency (%)		83.89 [70.95, 88.46]	82.48 [75.94, 88.82]	84.33 [77.20, 89.58]	0.64
Sleep latency (min)		15.71 [7.71, 18.75]	14.29 [9.14, 22.54]	14.57 [10.00, 21.71]	0.664

Categorical variables are displayed as n (%). Continuous variables are displayed as median [interquartile range].

Parameters were evaluated in a univariate analysis.

Nonparametric variables were compared using the Kruskal-Wallis test in the additive model and the Mann-Whitney U test in the recessive model.

Pearson chi-square test was used for categorical parameters.

Abbreviations: BMI, body mass index; OSA, obstructive sleep apnea; PSQIG, The Pittsburgh Sleep Quality Index Global score; SNP, single nucleotide polymorphism.

Next, a recessive model was used to perform inter-genotype comparisons for individual PSQI items related to OSA (Table 4). Subjective sleep efficiency (ratio of actual sleep time/bedtime in the past month) was significantly lower in the A genotype of rs2254298 (*P* = .013). The questionnaire asking about the frequency of symptoms associated with OSA, the frequency of sleep difficulty due to breathlessness assessed by Q5d (*P* = .00045), and coughing and loud snoring assessed by Q5e (*P* = .0089) revealed that they were both significantly higher in the A genotype (Table 4). To account for multiple comparisons across these 3 outcomes, Bonferroni correction was applied to control for the risk of type I errors. Even after applying the correction, the differences between genotypes remained statistically significant, with the corrected alpha set at 0.0167.

**Table 4.** Association of rs2254298 genotype with PSQI items related to OSA

Parameters		rs2254298 recessive model		
	Severity	A Genotype, n = 33	G Genotype, n = 272	*P* value
Subjective sleep efficiency		0.78 [0.40, 0.97]	0.92 [0.75, 1.00]	.013*^a^*
	0	28 (84.8)	265 (97.4)	.00045*^a^*
C5 Q5d. (%)	1	3 (9.1)	4 (1.5)	
Cannot breathe comfortably	2	0 (0.0)	2 (0.7)	
	3	2 (6.1)	1 (0.4)	
	0	27 (81.8)	256 (94.1)	.0089*^a^*
C5 Q5e. (%)	1	3 (9.1)	11 (4.0)	
Cough or snore loudly	2	2 (6.1)	4 (1.5)	
	3	1 (3.0)	1 (0.4)	

Categorical variables are displayed as n (%). Continuous variables are displayed as median [interquartile range].

Nonparametric variables were compared using the Mann-Whitney U test.

^*a*^Significant difference (corrected α = 0.0167, Bonferroni procedure).

C5 asks *how often have you had trouble sleeping during the past month* for each reason.

The scores for each item of C5 are as follows. 0, not during the past month; 1, less than once a week; 2, once or twice a week; 3, three or more times a week.

**Figure 2.**
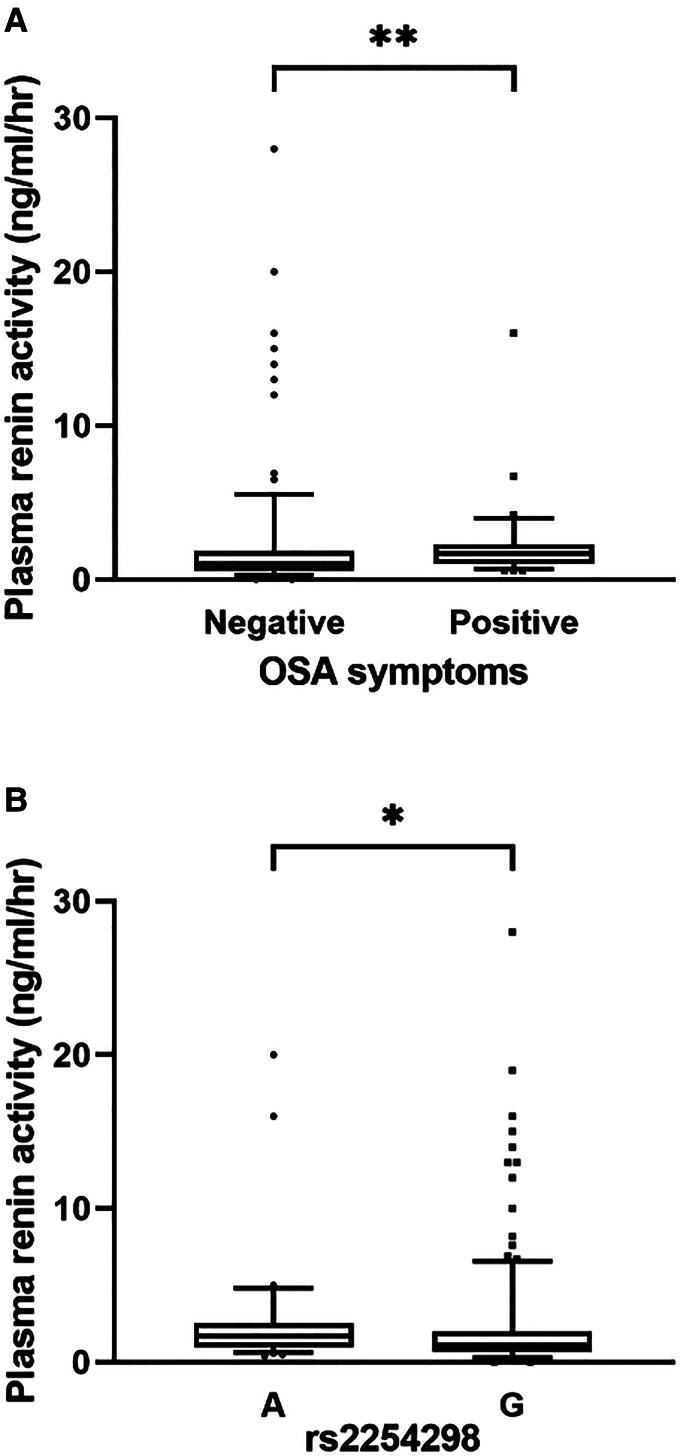
Association of the plasma renin activity with OSA symptoms and rs2254298 genotype. (A) Comparison of plasma renin activity with (n = 173) and without (n = 29) OSA symptoms. (B) Comparison of plasma renin activity between rs2254298 A genotype (n = 33) and rs2254298 G genotype participants (n = 272) (recessive model). **P < .01; *P < .05; Abbreviation: ns, not significant.

OSA activates the sympathetic activity [7], which can lead to increased plasma renin activity [56]. Indeed, plasma renin activity was significantly higher (*P* = .0025) in participants with OSA symptoms in our cohort (Fig. 2A). Plasma renin activity was significantly higher in the rs2254298 A genotype (*P* = .011, recessive model) than in the G genotype, supporting OSA symptom exacerbation in the A genotype (Fig. 2B).

In addition to OSA, chronic renal failure, chronic heart failure, and bronchial asthma are associated with paroxysmal nocturnal dyspnea [57-59]. Estimated GFR based on serum cystatin C (eGFRcys), N-terminal proBNP (NT-proBNP), and serum immunoglobulin E (IgE), which reflect the pathophysiology of these diseases, showed no significant differences between genotypes (Table 5).

**Table 5.** Association of rs2254298 genotype with activity indices of diseases that can cause breathlessness during sleep

rs2254298.recessive	A Genotype	G Genotype	*P* value
eGFRcys (mL/min/1.73 m^2^)	79.80 [65.95, 100.25]	82.20 [70.83, 94.30]	.826
Nonspecific IgE (IU/mL)	59.80 [17.50, 138.00]	62.35 [18.00, 163.50]	.77
NT-proBNP (pg/mL)	38.00 [22.50, 81.00]	42.00 [23.00, 66.00]	.963

Continuous variables are displayed as median [interquartile range].

Nonparametric variables were compared using the Mann-Whitney U test.

Abbreviations: eGFRcys, estimated GFR based on serum cystatin C; IgE, immunoglobulin E; NT-proBNP, N-terminal proBNP.

Although alcohol consumption is known to increase the frequency and duration of sleep apnea [60], no significant differences in alcohol consumption were observed between the rs2254298 genotypes (Table 3). Similarly, while obesity is a known risk factor for OSA [61], no significant BMI differences were observed between the rs2254298 genotypes (Table 3). We examined whether there was an interaction between rs2254298 genotype and alcohol intake or BMI with respect to OSA-related symptoms assessed by Q5d and Q5e of the PSQI. No significant interaction was observed between rs2254298 and alcohol consumption (Q5d, *P* = .086; Q5e,,*P* = .194). On the other hand, interaction analysis between rs2254298 and BMI (SNP × BMI interaction) showed a suggestive effect on OSA symptoms (Q5d; *P* = .356; Q5e, *P* = .035). However, as we simultaneously evaluated 2 outcomes (Q5d and Q5e), Bonferroni correction was applied to control for multiple comparisons, and the significance threshold was set at 0.025. None of the interactions reached this corrected significance level. Specifically, the effect of the rs2254298 genotype on OSA risk appears to decrease as BMI increases, although these results should be interpreted with caution due to the lack of statistical significance. Despite this potential interaction, multivariable analysis adjusting for BMI, sex, and age revealed that the OXTR rs2254298 A genotype remained an independent risk factor for increased sleep difficulty (items Q5d and Q5e; Table 6). Even after adjusting for smoking and alcohol intake, which can be problematic when extrapolating results from this population with lower smoking prevalence and potentially more health-conscious behaviors to the general population, the OXTR rs2254298 A genotype remained a significant risk factor for OSA-related symptoms (Table 6).

**Table 6.** Multivariable analysis of the association between the rs2254298 genotype and OSA-related PSQI items

Factors	Odds ratio	*P* value
Q5d		
Model 1: Adjusted for Age, Sex, and BMI	6.62 (1.79-23.04)	.0031*^a^*
Model 2: Model 1+ Alcohol intake	6.10 (1.18-30.49)	.025*^a^*
Model 3: Model 1+ Smoking habits	6.76 (1.82-23.58)	.0029*^a^*
Model 4: Model 1+ Drinking habits	6.85 (1.85-23.98)	.00259*^a^*
Q5e		
Model 1: Adjusted for Age, Sex, and BMI	3.75 (1.23-10.24)	.013*^a^*
Model 2: Model 1+ Alcohol intake	3.83 (1.02-13.07)	.036*^a^*
Model 3: Model 1+ Smoking habits	3.68 (1.21-10.07)	.015*^a^*
Model 4: Model 1+ Drinking habits	3.65 (1.20-10.02)	.015*^a^*

Multivariable *P* values were from ordinal logistic regression analyses.

^*a*^Significant difference (*P* < .05).

To examine the contribution of *OXTR* polymorphism to OSA by population, the rs2254298 A allele frequency among populations was compared, showing that the A allele frequency was higher in the Japanese and East Asian populations than in the European and other populations (Table 7).

**Table 7.** Interpopulation comparison of rs2254298 SNP A allele frequency

	Shika-cohort	1000 Genomes Project				
	Japanese	East Asia	South Asia	European	American	African
A allele frequency	0.285	0.356	0.105	0.109	0.223	0.249

**Figure 3.**
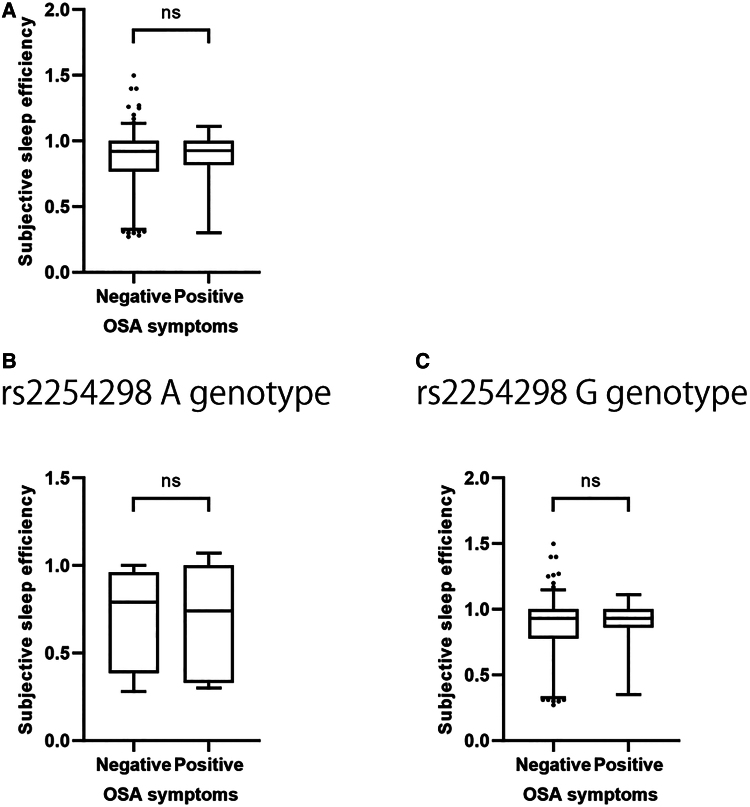
Association of subjective sleep efficiency with OSA symptoms. A, Comparison of subjective sleep efficiency between participants with (n = 189) and without (n = 36) OSA symptoms for all participants. B, Comparison of subjective sleep efficiency between participants with (n = 16) and without (n = 7) OSA symptoms in the rs2254298 A genotype (recessive model). C, Comparison of subjective sleep efficiency between participants with (n = 173) and without (n = 29) OSA symptoms in the rs2254298 G genotype (recessive model). Abbreviation: ns, not significant.

Regarding the cause of decreased subjective sleep efficiency in the A genotype, OSA symptoms might contribute to decreased subjective sleep efficiency; however, no difference in subjective sleep efficiency was observed between participants with and without OSA symptoms (*P* = .43) (Fig. 3A). In addition, the same analysis was performed for each rs225428 genotype, and no significant differences in subjective sleep efficiency were observed between the groups with and without OSA symptoms in either the A genotype or G genotype of the recessive model (*P* = .737, *P* = .365, respectively) (Fig. 3B and 3C).

## Discussion

The present study comprehensively evaluated the *OXTR* polymorphism, sleep quality assessment based on questionnaires, and sleep measurements. The *OXTR* rs2254298 A allele was associated with the frequency of OSA symptoms and subjective sleep efficiency.

By what mechanism is OXT signaling involved in OSA? Ventilatory drives from the medullary respiratory center operate on the diaphragm and other respiratory muscles, as well as the hypoglossal nerve, which innervates the genioglossus muscle, a group of muscles involved in opening the upper airway. The instability of this ventilatory drive from the medullary respiratory center is present in OSA pathology [11]. In addition, neuromuscular electrical stimulation of the hypoglossal nerve has been used as a potential alternative to positive airway pressure therapy, suggesting the importance of upper airway collapse due to decreased genioglossus muscle activity in the OSA pathogenesis [10, 62]. Paraventricular nucleus–derived OXTnergic neurons project to the rostral ventrolateral medulla region and phrenic nucleus and exert respiratory output via diaphragmatic movements [63]. Furthermore, a recent animal study reported that OXT receptors are also abundant in hypoglossal motoneurons in the medulla and that OXT administration stimulates respiratory-related tongue muscle activity, which in turn facilitates the opening of the upper airway [64]. Indeed, clinical studies in patients with OSA have confirmed that intranasal OXT treatment significantly reduced the duration of obstructive events and the incidence of oxygen desaturation and bradycardia and increased respiratory rate during the nonobstructive period, consistent with the supposed physiological effects of OXTergic neurons [26]. Respiratory control system abnormalities have been implicated in the familial clustering of OSA; however, its specific genetic predisposition has not yet been determined [63, 65]. Combined with our findings, *OXTR* could be a genetic predisposition involved in the OSA pathogenesis via modulation of the respiratory control system, including the phrenic nerve and the hypoglossal nerve.

Mice deficient in the *OXTR* gene exhibit an obese phenotype due to impaired thermogenesis [66]. Since obesity is a typical risk factor for OSA [61] and is strongly influenced by genetic predisposition [67], the association between obesity and *OXTR* polymorphisms was also examined. However, no significant differences in BMI were observed among the rs2254298 genotypes, indicating that rs2254298 may not be directly associated with BMI in this population.

In the current study, the rs2254298 A genotype, the genetic predisposition for OSA, was not associated with objective sleep efficiency as measured by actigraph but with lower subjective sleep efficiency as assessed by the PSQI. Conversely, our findings indicate that reduced subjective sleep efficiency is not explicitly associated with OSA symptoms and that reduced subjective sleep efficiency in individuals with the rs2254298 A genotype may also be independent of OSA symptoms. Although the exact mechanism of subjective–objective sleep discrepancy as observed in the OXTR rs2254298 A genotype is not yet understood [68], considering that *OXTR* SNPs are associated with negative-emotion reactivity under a stressful context [69], A allele carriers may perceive stresses such as sleep latency and awakenings as more significant and underestimate actual sleep duration.

Regarding the ethnic clustering of OSA, studies comparing the association between OSA and population within the same cohort have found higher ORs for OSA in Asians compared with European-origin individuals when adjusted for age and BMI [70, 71]. Anatomical issues such as the distribution of fat deposition and craniofacial structure have been assumed to account for these ethnic differences [72]. Still, the influence of specific genetic predisposition differences and nonanatomical factors has not been evident. The A allele of rs2254298, the risk allele identified in this study, is particularly enriched in Japanese and East Asians compared with other populations. Therefore, this risk allele could explain the genetic background of the higher risk of OSA in Asians by mechanisms other than anatomic factors.

The present study is the first to consider *OXTR* as a genetic predisposition of OSA. However, the following limitations of this study are inevitable: first, familial clustering has also been reported to exist in the anatomical characteristics of maxillofacial morphology in patients with OSA, and 5 loci among the genes identified in the GWAS of OSA are associated with facial morphological characteristics [73]. In the present study, data on maxillofacial morphological characteristics were not obtained, making it difficult to evaluate the association between *OXTR* gene polymorphisms and maxillofacial morphology. Second, although sleep apnea status was estimated in the present study based on subjective symptoms and hypoxia-responsive sympathetic nerve activity, a follow-up study is needed to further improve the design by adding polysomnographic indices, which are essential for the definitive diagnosis of OSA. The third limitation of our study is the assessment of alcohol consumption using the self-reported Japanese adaptation of the BDHQ. Self-reported measures can introduce biases, such as underreporting of alcohol consumption. Additionally, the BDHQ was not administered to all participants due to differences in implementation by year, which can be considered a form of random allocation. There were no significant differences in rs2254298 genotype, alcohol consumption habits, OSA symptom-related indicators, age, or gender between those who completed the BDHQ and those who did not (data not shown). Therefore, we believe that the results of our multivariable analyses, adjusted for alcohol consumption, can be generalized to the overall study population. A fourth limitation of this study was the low smoking rate among participants with sleep/actigraphy data. This may have resulted in selection bias. This group had fewer smokers compared to the overall population, suggesting that the overall risk of OSA may be lower due to greater health awareness. Therefore, caution should be used in generalizing the results. However, the association between rs2254298 genotype and OSA symptoms remained significant even after adjusting for potential confounders such as smoking and alcohol consumption, which are problematic in health-oriented populations. This suggests that the genotype-phenotype relationship is robust and potentially generalizable despite initial selection bias.

In conclusion, OXTR polymorphism rs2254298 was identified as a genetic polymorphism associated with OSA symptoms, subjective sleep efficiency, and sympathetic activity, independently of alcohol intake and weight. However, it is important to note that rs2254298 may not be the direct causal variant, but it could be in linkage disequilibrium with another causal variant. Further studies are needed to explore the true causal variants. The A allele of rs2254298 may contribute to the increased risk of OSA in Asians compared with other ethnic groups. The current study highlights the potential of OXT as a treatment for OSA, for which there is still no FDA-approved treatment.

## Data Availability

The datasets analyzed in the current study are not publicly available but are available from the corresponding authors upon reasonable request.

## Clinical Trials Registration

This study was approved by the Ethics Committee for Human Studies at Kanazawa University Hospital (1491, 2016-376) and performed following the principles outlined in the Declaration of Helsinki.

## Abbreviations

BDHQ: brief-type self-administered diet history questionnaire
BMI: body mass index
eGFRcys: estimated glomerular filtration rate based on serum cystatin C
GWAS: genome-wide association study
IgE: immunoglobulin E
NT-proBNP: N-terminal pro-brain-type natriuretic peptide
OSA: obstructive sleep apnea
OXT: oxytocin
OXTR: oxytocin receptor
PSQI: Pittsburgh Sleep Quality Index
SNP: single nucleotide polymorphism
